# Intra- and Extra-Articular Deformity of Lower Limb: Tibial Condylar Valgus Osteotomy (TCVO) and Distal Tibial Oblique Osteotomy (DTOO) for Reconstruction of Joint Congruency

**DOI:** 10.1155/2019/8605674

**Published:** 2019-02-18

**Authors:** Y. Watanabe, N. Takenaka, K. Kinugasa, T. Matsushita, T. Teramoto

**Affiliations:** ^1^Department of Orthopaedic Surgery, Teikyo University School of Medicine, Tokyo, Japan; ^2^Department of Traumatology Fukushima Medical University, Trauma and Reconstruction Center, Southern Tohoku General Hospital, Fukushima, Japan; ^3^Department of Orthopaedic Surgery, Chikamori Hospital, Kochi, Japan

## Abstract

Osteotomies are the established surgical procedure for the deformity of the lower limb induced by osteoarthritis (OA) of the knee and ankle. Closed-wedge (CW) and open-wedge (OW) high tibial osteotomy (HTO) are extra-articular surgery, which aim to shift the mechanical axis from medial to slightly lateral and reduce the overload in the medial compartment of the varus deformed knee by extra-articular correction. However, varus deformity of the knee with the teeter effect, which could be accompanied with subluxation and thrust due to the medial-lateral soft tissue imbalance, is not resolved only by the shift of mechanical axis. The depression of the medial tibia plateau, so-called pagoda deformity, is the intra-articular deformity, which could potentially cause the teeter effect and involves intra-articular incongruency. In such case, the osteotomy with novel concept should be developed to overcome the issues, both the imbalance of soft tissue and intra-articular deformity. Tibial condylar valgus osteotomy (TCVO) is an intra-articular osteotomy, which improves the joint congruency of the medial-compartment knee OA with subluxation and/or intra-articular deformity and also provides better joint stability. A similar argument is raised in the treatment of the ankle OA. Low tibial osteotomy (LTO) is an extra-articular surgery to correct malalignment of lower leg. Distal tibial oblique osteotomy (DTOO) is a novel surgery to improve the bony congruency of the ankle OA. In DTOO, the distal tibia is cut obliquely from the proximal medial to the distal lateral in the coronal plane and towards the center of the tibiofibular joint to improve the bony congruency of the ankle joint. Tibial condylar valgus osteotomy (TCVO) and distal tibial oblique osteotomy (DTOO) can correct intra-articular deformity of knee and ankle, respectively. The rationale and indication of TCVO and DTOO for the treatment of the lower limb by reconstructing the joint congruency are discussed.

## 1. Introduction

Deformity of the lower limb induced by osteoarthritis of the knee or ankle causes the adverse effect on daily living due to gait disturbance, which could cause abnormal posture, as well as local problem such as pain. Various surgical procedures have been performed such as femoral, tibial osteotomies, and hip and knee arthroplasties to overcome these issues. Osteotomies are the established surgical options for the treatment of lower limb.

Osteoarthritis of the knee is a representative disease, which involves malalignment of the lower limb with the joint degeneration. Arthroplasty of the knee, involving total knee arthroplasty (TKA), unicompartmental knee arthroplasty (UKA), is established as surgical procedures with good clinical results [1–3]. Around knee osteotomies (AKO) have also been performed extensively for the treatment of this disease and have shown the good clinical outcomes by correcting the alignment of lower limb. High tibial osteotomy (HTO) is performed extensively, which aims to correct the varus knee arthrosis. Many studies demonstrated the good results by cutting the proximal tibia as lateral closed-wedge high tibial valgus osteotomy (CWHTO) or dome valgus osteotomy. Medial open-wedge high tibial valgus osteotomy (OWHTO) has also been developed for the correction of the varus deformed knee and extensively spread by the improvement in surgical technique, fixation devices, and patient selection with fewer complications [4–7]. Hybrid closed-wedge osteotomy, combining OWHTO and CWHTO, has currently performed with disadvantages of traditional CWHTO including lateral-offset, loss of the large bone block below the lateral tibial plateau, and discrepancies in the leg length [8]. High tibial valgus osteotomy is an established surgical procedure to correct varus malalignment in patients with medial-compartment OA of the knee; however, the deformity center of the malalignment of lower limb varies in each case. Distal femoral osteotomy (DFO) has also been performed for the patient with osteoarthritis with the deformity in distal femur and increasingly performed alone or combination with HTO with the improvement in such as surgical technique and fixation devices same as development of medial opening wedge HTO.

The concept of traditional HTO has been the extra-articular correction of the alignment, aiming at the reduction of the overload in the medial compartment of the varus deformed knee by shifting the mechanical axis from medial to slightly lateral. On the other hand, deformities of the lower limb are affected by several factors. The bony deformity and soft tissue balance are the different issues relating to the malalignment. Varus deformity in OA of the knee without excessed medial loosening of soft tissue could be corrected by usual HTO, which is the extra-articular correction with transfer of the mechanical axis. However, the varus deformity of the knee with the teeter effect, which is the medial-lateral soft tissue imbalance, is not resolved only by the shift of mechanical axis to lateral. The depression of the medial tibia plateau, so-called pagoda deformity, is the intra-articular deformity, which could cause intra-articular incongruency and the teeter effect. In such case, the osteotomy with novel concept should be developed to overcome the issues, both the imbalance of soft tissue and intra-articular deformity; otherwise the relief of the pain and disability would not be achieved.

For the treatment of osteoarthritis of ankle joint, the same argument exists as the correction of the varus knee. Low tibial osteotomy (LTO) for osteoarthritis of ankle joint has been performed to change the load distribution on the ankle joint by changing the extra-articular alignment [9, 10]. However, if instability of the talotibial joint exists, novel surgical concept should be induced for the treatment of the osteoarthritis of the ankle joint. Distal tibial oblique osteotomy (DTOO) developed is an operation to improve the bony congruency of the ankle joint by the oblique osteotomy from the proximal medial to the distal lateral in the coronal plane and towards the center of the tibiofibular joint to realign the bony congruency of the ankle joint [11–13].

Considering the correction of lower limb alignment, regardless of cause of mal-alignment, such as post-traumatic, degenerative, or congenital, we need to consider two kinds of deformities: extra-articular and intra-articular deformity. Extra-articular deformity can be discussed under static loading condition, and intra-articular deformity should be discussed under dynamic loading condition.

Extra-articular deformity in frontal plane is evaluated by the measurement of the standing long-leg AP view. Theoretically, there are 728 kinds of extra-articular deformity, and these deformities can be accurately corrected by using hexapod system. Evaluation of intra-articular deformity should be evaluated by dynamic motion of the contours of the bone facing in the joint. Tibial condylar valgus osteotomy (TCVO) and distal tibial oblique osteotomy (DTOO) can correct intra-articular deformity of knee and ankle, respectively.

## 2. Extra-Articular Deformity and Lower Limb Alignment

**Figure 1:**
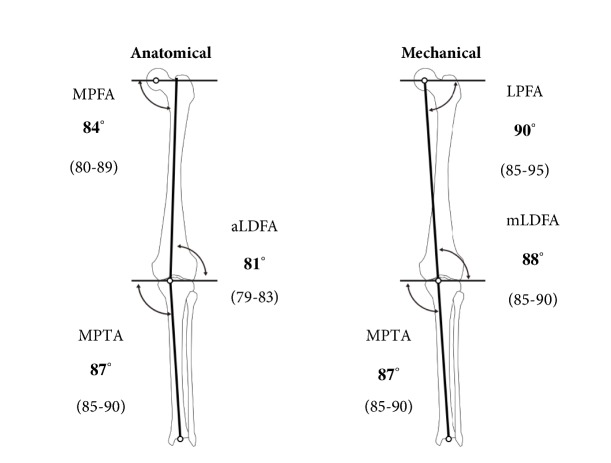
Normal lower limb alignment by Paley D.

**Figure 2:**
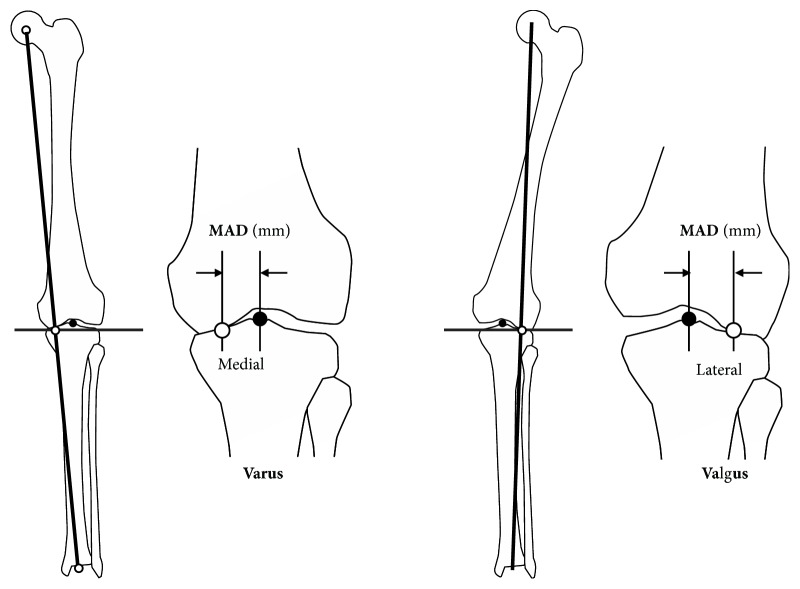
Mechanical axis deviation (MAD) is the perpendicular distance between the mechanical axis of the lower extremity and the center of the knee joint.

Traditionally the degree of extra-articular deformity of the frontal plane has been expressed as the degree of deviation from the mechanical axis of the lower extremity [14] (Figure 1). The mechanical axis of the lower extremity is determined by drawing a line from the center of the femoral head to the center of the ankle joint. The distance between the mechanical axis line and the center of the knee in the frontal plane is called as the mechanical axial deviation (MAD) [15, 16] (Figure 2). Normally, the medial MAD is 1 mm to 15 mm.

**Figure 3:**
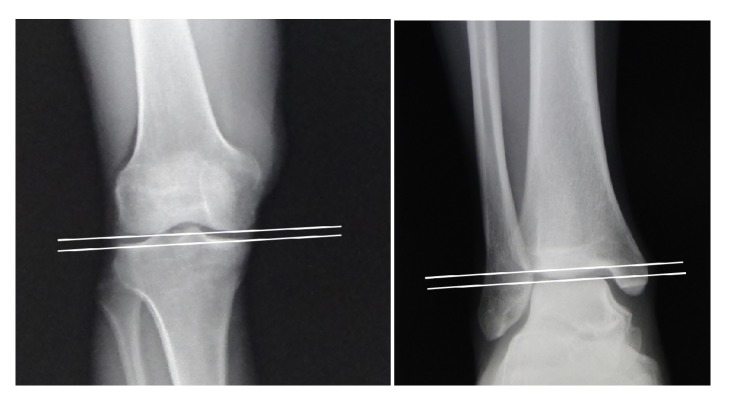
Joint line convergence angle (JLCA) for knee and ankle.

The frontal plane knee joint orientation line of the distal femur is drawn as a line tangential to the most distal point on the convexity of the two femoral condyles. The frontal plane knee joint line of the proximal tibia is drawn across the subchondral line of the two tibial plateaus. The angle formed between joint orientation lines on opposite sides of the same joint is called the joint line convergence angle (JLCA) [14]. The ankle JLCA is defined as the angle formed between the tibial joint line axis and the talar joint line axis (Figure 3). In the knee and ankle joints, each JLCA is measured as 0°-2° and -1°-1°, respectively.

The deviation from the normal of the lower extremity alignment can be evaluated by the joint orientation angles, such as MPTA, LDFA, LPDA, etc. measured with the full leg standing frontal X-ray image of the lower limb [14] (Figure 1). Malalignment occurs when the center of the joint does not lie close to mechanical axis line. The MAD is described as either medial (varus) or lateral (valgus) MADs (Figure 1).

Angular deformity has four types: varus/valgus deformity at the frontal plane and procurvatum/recurvatum deformity at the sagittal plane. Similarly, there are four kinds of deformities in translation (medial/lateral at the frontal plane, and anterior/posterior at the sagittal plane) and axial (internal/external rotation and shortening/elongation) deformities. Consequently, there are 728 patterns of extra-articular deformities.

**Figure 4:**
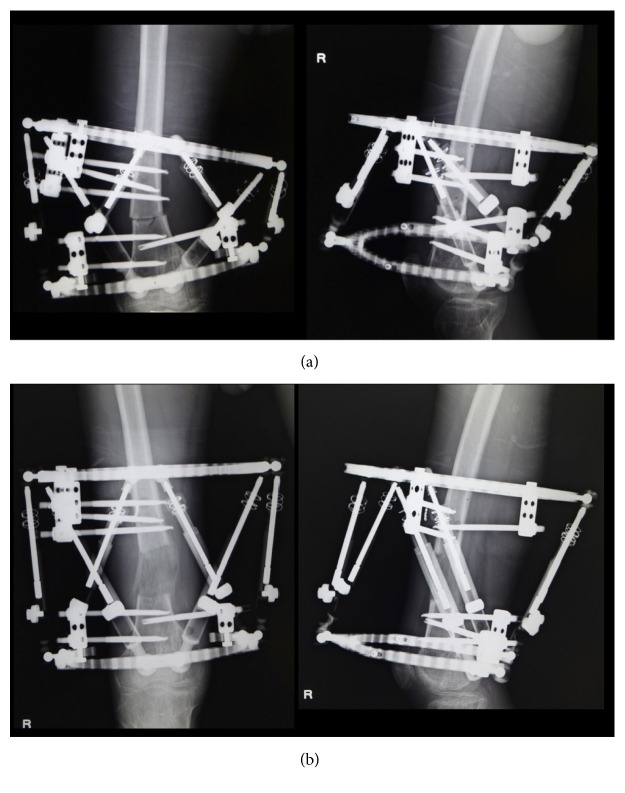
Reconstruction of multidirectional deformity by using the hexapod external fixator system. (a) Application of a hexapod external fixator; (b) completion of the reconstruction program.

**Figure 5:**
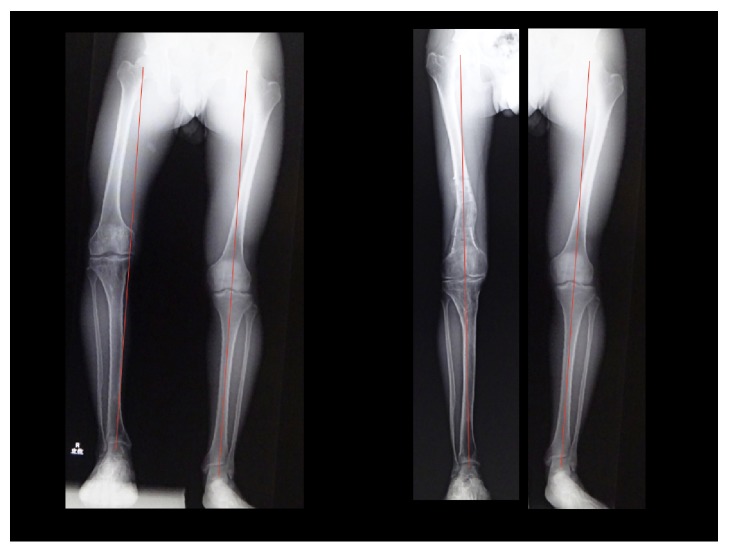
Correction of extra-articular deformity by using hexapod external fixator system. X-ray photos (a) before and (b) after reconstruction.

The angular deformity correction of lower limb can be achieved using hinges set on center of rotation of angulation (CORA) of the connecting rods by Ilizarov technique [14]. Currently used hexapod external fixator system can correct multi-directional extra-articular deformity accurately within the rage of measurement in units of 1 mm and 1° [17, 18] (Figures 4 and 5).

## 3. Intra-Articular Deformity and Joint Congruency

Intra-articular deformity means joint congruency. Joint congruency is defined as the fittings of two opposing joint surfaces as they relate to one another considering the spatial contour of each bone at their interface.

**Figure 6:**
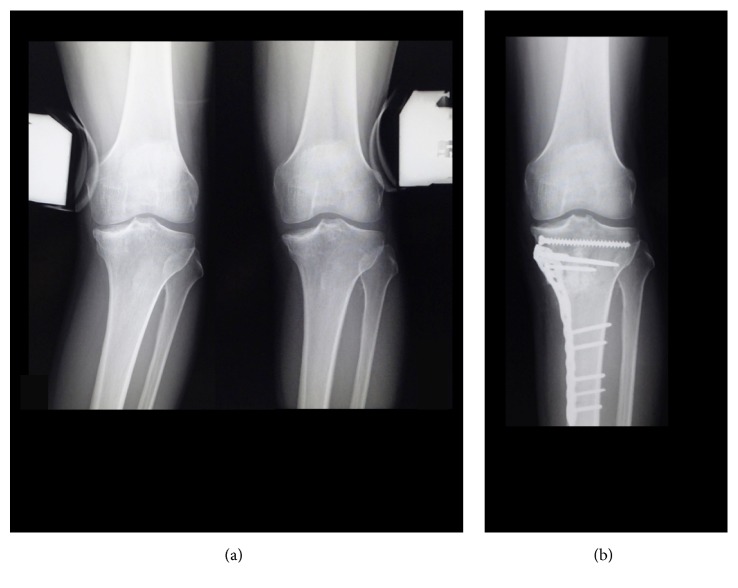
(a) Teeter effect due to intra-articular deformity after medial tibial plateau fracture. (b) After reconstruction by tibial condylar valgus osteotomy (TCVO).

In theory, joint congruency can be mainly explained by three factors: contours of bone, functions of ligament, and integrity of articular cartilage and meniscus. Incongruency of the joint immediately causes joint instability or abnormal joint kinematics resulting in the secondary osteoarthritis in the future. For example, mal-union of tibial plateau fracture and/or knee ligament injury such as anterior cruciate ligament and/or collateral ligaments causes easily knee instability and secondary osteoarthritis of the involved knee with joint incongruency (Figure 6(a)).

Two primary biomechanical purposes of the articular cartilage are lubrication and shock absorption. The articular cartilage provides a smooth lubricated surface for low friction articulation and acts as a remarkably efficient shock absorber [19, 20].

From the view point of joint congruency, it is considered that the effect of articular cartilage as the shock absorber is not large because the thickness of articular cartilage is thin as 1 – 4 mm. Rather in the cartilaginous element, the menisci may be more important because they have a large effect of filling the gap of the joint [21]. However, cartilage and meniscus do not function enough when the intra-articular bony deformity occurred. Furthermore, ligament function is effective only when the contour of bone is not impaired. Therefore, joint congruency due to the contour of bone plays the most important role.

## 4. TCVO (Tibial Condylar Valgus Osteotomy) and HTO (High Tibial Osteotomy)

High tibial osteotomy (HTO) is intended to transfer the mechanical axis from medial to slightly lateral to the midline of the knee to decrease the load and subsequently delay osteoarthritis (OA) [22–24]. There are discussions about which alignment is best in HTO but we think that it should be discussed first whether accurate lower extremity alignment can be obtained by the procedures reported [25–31]. If we aim to acquire more accurate lower limb alignment, we should use hexapod external fixator which can theoretically correct extra-articular alignment accurately within the range of measurements in units of 1 mm and 1°.

**Figure 7:**
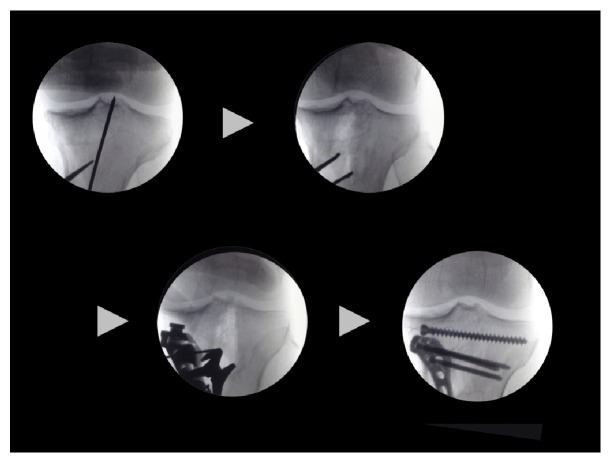
Tibial condylar valgus osteotomy (TCVO).

**Figure 8:**
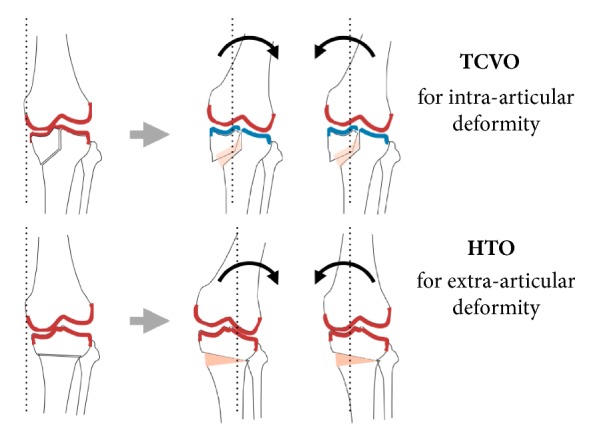
TCVO can reconstruct the joint congruency and alignment, however, HTO change only static lower limb alignment. Teeter effect cannot be improved by HTO.

On the other hand, tibial condylar valgus osteotomy (TCVO) developed by Chiba G et al. in 1989 and published in 1994 is an operation that corrects intra-articular deformity [32, 33] (Figures 6(b) and 7). The main strategy of this surgery is to obtain joint stability and congruency, which is totally different from that of HTO to correct malalignment [32, 33] (Figure 8). TCVO is an operation to acquire the bony joint stability between femur and tibia by the “L” shaped osteotomy of medial tibial condyle and spreading it [32, 33] (Figure 7). It is determined how much the medial tibial condyle is spread by judging joint stability under fluoroscopic dynamic stress test including the rotational stability. However, performing TCVO on a common varus knee improves lower limb alignment (Figure 8). The correction of alignment is an accessory benefit, which is subsequently brought by TCVO. It should not be misunderstood that TCVO is one of HTO subtypes and be aware that the main concept of TCVO is to obtain the joint stability and congruency.

## 5. Distal Tibial Oblique Osteotomy (DTOO) and Low Tibial Osteotomy (LTO)

**Figure 9:**
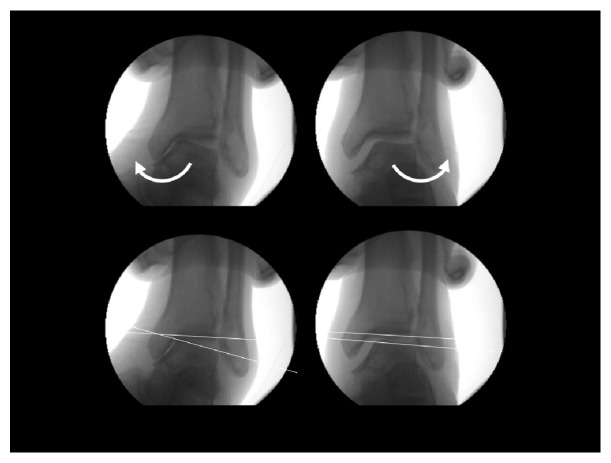
Instability of talotibial joint.

The same argument holds for distal tibial oblique osteotomy (DTOO) and low tibial osteotomy (LTO) for osteoarthritis of ankle joint. LTO is an operation of an attempt to change the load distribution on the ankle joint by changing the extra-articular alignment. However, if instability of the talo-tibial joint exists (Figure 9), improvement of the joint function by LTO depends on luck by self-centering function of the talus.

**Figure 10:**
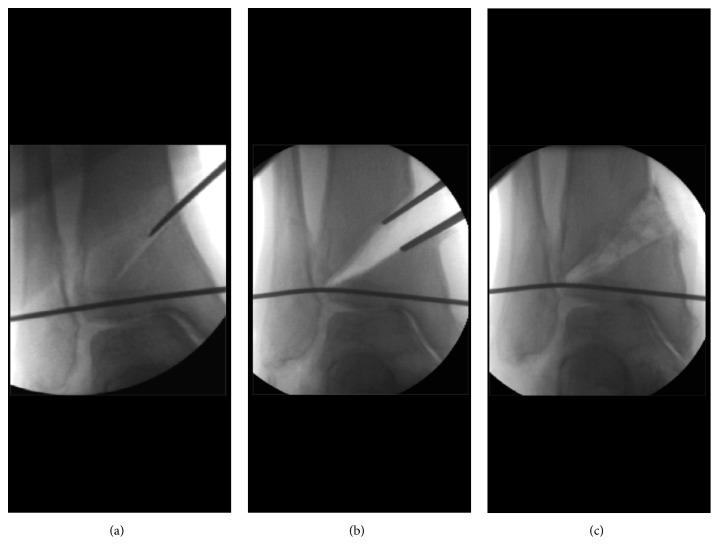
Distal tibial oblique osteotomy (DTOO).

On the other hand, DTOO developed by Teramoto in 1994 is an operation to improve the bony congruency of the ankle joint [11]. DTOO consists of oblique osteotomy from the proximal medial to the distal lateral in the coronal plane and towards the center of the tibiofibular joint and then widening of the osteotomy site to realign the bony congruency of the ankle joint (Figure 10). DTOO is a surgical technique to realign the ankle joint surface. The shape of the ankle joint surface is changed by cutting and tilting the tibial plafond without osteotomy of the fibula. The inclination of the distal tibial articular surface with respect to the tibial axis is altered, with associated improvement in ankle stability [11–13]. The oblique osteotomy site is spread until the lateral talar articular surface contact with the lateral fibular malleolus. The ankle mortise congruency is restored since medial and lateral articular gutters on either side of the talar body are symmetrical which induces the increase of contact area of the talar articular surface with the tibia and fibula. The increased contact area, dispersed load pressure across the ankle joint, and decrease in the load pressure per unit area applied to the ankle joint would improve congruency between the articular surfaces of the talar dome and distal tibial plafond and the medial and lateral gutters of the ankle mortise would improve ankle stability.

## 6. Discussion

Although osteotomies are effective in managing malalignment of knee and ankle arthrosis, determination regarding the type of osteotomy should be based on the pathology in each patient. The cause of deformity of the knee varies, which involves intra- and extra-articular or the combination of them. Center of the deformity may also vary. Soft tissue balance should be another critical issue for the selection of optimal osteotomy type. Traditional HTO should be an optimal strategy to overcome the malalignment in OA of the knee; however, the excessed medial-lateral soft tissue imbalance and intra-articular deformity are not solved by this surgical procedure. Tibial condylar valgus osteotomy (TCVO) should be an optimal option to overcome these issues that improves intra-articular deformity followed by acquisition of medial-lateral stability of the knee, not lower limb alignment. Although LTO is the good treatment for the deformity in OA of ankle by the extra-articular correction of alignment, treatment for incongruency and instability for OA in ankle is the remaining issue of this surgical technique. Distal tibial oblique osteotomy improves the bony congruency of the ankle joint by the oblique osteotomy.

Tibial condylar valgus osteotomy and DTOO that correct intra-articular deformity of knee and ankle are the optimal osteotomies for OA with intra-articular deformity and instability.

## References

[1] Aujla R. S., Esler C. N. (2017). Total knee arthroplasty for osteoarthritis in patients less than fifty-five years of age: a systematic review. *The Journal of Arthroplasty*.

[2] Siman H., Kamath A. F., Carrillo N., Harmsen W. S., Pagnano M. W., Sierra R. J. (2017). Unicompartmental knee arthroplasty vs total knee arthroplasty for medial compartment arthritis in patients older than 75 years: comparable reoperation, revision, and complication rates. *The Journal of Arthroplasty*.

[3] Franceschetti E., Torre G., Palumbo A. (2017). No difference between cemented and cementless total knee arthroplasty in young patients: a review of the evidence. *Knee Surgery, Sports Traumatology, Arthroscopy*.

[4] Duivenvoorden T., Brouwer R. W., Baan A. (2014). Comparison of closing-wedge and opening-wedge high tibial osteotomy for medial compartment osteoarthritis of the knee: a randomized controlled trial with a six-year follow-up. *The Journal of Bone & Joint Surgery—American Volume*.

[5] Floerkemeier S., Staubli A. E., Schroeter S., Goldhahn S., Lobenhoffer P. (2013). Outcome after high tibial open-wedge osteotomy: A retrospective evaluation of 533 patients. *Knee Surgery, Sports Traumatology, Arthroscopy*.

[6] Hui C., Salmon L. J., Kok A. (2011). Long-term survival of high tibial osteotomy for medial compartment osteoarthritis of the knee. *The American Journal of Sports Medicine*.

[7] McNamara I., Birmingham T. B., Fowler P. J., Giffin J. R. (2013). High tibial osteotomy: evolution of research and clinical applications-a canadian experience. *Knee Surgery, Sports Traumatology, Arthroscopy*.

[8] Takeuchi R., Ishikawa H., Miyasaka Y., Sasaki Y., Kuniya T., Tsukahara S. (2014). A novel closed-wedge high tibial osteotomy procedure to treat osteoarthritis of the knee: hybrid technique and rehabilitation measures. *Arthroscopy Techniques*.

[9] Takakura Y., Tanaka Y., Kumai T., Tamai S. (1995). Low tibial osteotomy for osteoarthritis of the ankle. Results of a new operation in 18 patients. *The Journal of Bone & Joint Surgery (British Volume)*.

[10] Tanaka Y., Takakura Y., Hayashi K., Taniguchi A., Kumai T., Sugimoto K. (2006). Low tibial osteotomy for varus-type osteoarthritis of the ankle. *The Journal of Bone & Joint Surgery (British Volume)*.

[11] Teramoto T., Harada S., Takaki M. (2018). The Teramoto distal tibial oblique osteotomy (DTOO): surgical technique and applicability for ankle osteoarthritis with varus deformity. *Strategies in Trauma and Limb Reconstruction*.

[12] Teramoto T., Tasiro K., Ootsuka K., Takaki M., Makino Y., Asahara T. (2009). The changes in the instability of the ankle joint after distal tibial oblique osteotomy performed for the treatment of osteoarthritis of the ankle joint. *Journal of Japanese Association of External Fixation and Limb Lengthening*.

[13] Teramoto T., Harada S., Takaki M. (2014). Operative methods of arthroplasty used by intra-articular osteotomy of the knee joint and ankle joint. *Journal of Japanese Association of External Fixation and Limb Lengthening*.

[14] Paley D. (2002). *Principles of Deformity Correction*.

[15] Burghardt R. D., Paley D., Specht S. C., Herzenberg J. E. (2012). The effect on mechanical axis deviation of femoral lengthening with an intramedullary telescopic nail. *The Journal of Bone & Joint Surgery (British Volume)*.

[16] Gordon J. E., Chen R. C., Dobbs M. B., Luhmann S. J., Rich M. M., Schoenecker P. L. (2009). Interobserver and intraobserver reliability in the evaluation of mechanical axis deviation. *Journal of Pediatric Orthopaedics*.

[17] Wilczek J. L., LaPorta G. A. (2018). The evolution of limb deformity: what has changed over the past ten years?. *Clinics in Podiatric Medicine and Surgery*.

[18] Arvesen J. E., Tracy Watson J., Israel H. (2017). Effectiveness of treatment for distal tibial nonunions with associated complex deformities using a hexapod external fixator. *Journal of Orthopaedic Trauma*.

[19] Sophia Fox A. J., Bedi A., Rodeo S. A. (2009). The basic science of articular cartilage: structure, composition, and function. *Sports Health*.

[20] Mow V. C., Holmes M. H., Lai W. M. (1984). Fluid transport and mechanical properties of articular cartilage: a review. *Journal of Biomechanics*.

[21] Fukuda Y., Takai S., Yoshino N. (2000). Impact load transmission of the knee joint-influence of leg alignment and the role of meniscus and articular cartilage. *Clinical Biomechanics*.

[22] Maquet P. (1985). The treatment of choice in osteoarthritis of the knee. *Clinical Orthopaedics and Related Research*.

[23] Coventry M. B. (1973). Osteotomy about the knee for degenerative and rheumatoid arthritis. *The Journal of Bone & Joint Surgery*.

[24] Akamatsu Y., Koshino T., Saito T., Wada J. (1997). Changes in osteosclerosis of the osteoarthritic knee after high tibial osteotomy. *Clinical Orthopaedics and Related Research*.

[25] Ivarsson I., Myrnerts R., Gillquist J. (1990). High tibial osteotomy for medial osteoarthritis of the knee. A 5 to 7 and an 11 to 13 year follow-up. *The Journal of Bone & Joint Surgery (British Volume)*.

[26] Hernigou P., Medevielle D., Debeyre J., Goutallier D. (1987). Proximal tibial osteotomy for osteoarthritis with varus deformity. A ten to thirteen-year follow-up study. *The Journal of Bone & Joint Surgery*.

[27] Coventry M. B. (1985). Upper tibial osteotomy for osteoarthritis. *The Journal of Bone & Joint Surgery*.

[28] Engel G. M., Lippert 3rd F. G. (1981). Valgus tibial osteotomy: avoiding the pitfalls. *Clinical Orthopaedics and Related Research*.

[29] Kettelkamp D. B., Chao E. Y. (1972). A method for quantitative analysis of medial and lateral compression forces at the knee during standing. *Clinical Orthopaedics and Related Research*.

[30] Koshino T., Morii T., Wada J., Saito H., Ozawa N., Noyori K. (1989). High tibial osteotomy with fixation by a blade plate for medial compartment osteoarthritis of the knee. *Orthopedic Clinics of North America*.

[31] Myrnerts R. (1980). Optimal correction in high tibial osteotomy for varus deformity. *Acta Orthopaedica*.

[32] Chiba G. (1994). Tibial condylar valgus osteotomy for osteoarthritis of knee. *OS Now*.

[33] Teramoto T. (2015). Controversy of high tibial osteotomy. *Journal of Limb Lengthening & Reconstruction*.

